# The Use of a Decision Support System in Swedish Pharmacies to Identify Potential Drug-Related Problems—Effects of a National Intervention Focused on Reviewing Elderly Patients’ Prescriptions

**DOI:** 10.3390/pharmacy8030118

**Published:** 2020-07-13

**Authors:** Tora Hammar, Lina Hellström, Lisa Ericson

**Affiliations:** 1 The eHealth Institute, Department of Medicine and Optometry, Linnaeus University, 391 82 Kalmar, Sweden; lina.hellstrom@regionkalmar.se; 2 Pharmaceutical Department, Region Kalmar County, 391 85 Kalmar, Sweden; 3 The Swedish eHealth Agency, 391 29 Kalmar, Sweden; lisa.ericson@ehalsomyndigheten.se

**Keywords:** clinical decision support system, pharmacy, drug-related problems, interrupted time series analysis, questionnaire

## Abstract

In pharmacies in Sweden, a clinical decision support system called Electronic Expert Support (EES) is available to analyse patients’ prescriptions for potential drug-related problems. A nationwide intervention was performed in 2018 among all Swedish pharmacy chains to increase the use of EES among patients 75 years or older. The aim of this research was to study the use of EES in connection with the national intervention in order to describe any effects of the intervention, to understand how pharmacists use EES and to identify any barriers and facilitators for the use of EES by pharmacists for elderly patients. Data on the number and categories of EES analyses, alerts, resolved alerts and active pharmacies was provided by the Swedish eHealth Agency. The effects of the intervention were analysed using interrupted time series regression. A web-based questionnaire comprising 20 questions was sent to 1500 pharmacists randomly selected from all pharmacies in Sweden. The study shows that pharmacists use and appreciate EES and that the national intervention had a clear effect during the week of the intervention and seems to have contributed to a faster increase in pharmacists’ use of EES during the year to follow. The study also identified several issues or barriers for using EES.

## 1. Introduction

Medication is an essential part of health care, and appropriate treatments with medications can cure and prevent many conditions [1]. However, drug-related problems (DRPs) are frequent and cause suffering for patients and lead to substantial costs for society [2,3,4]. Medication treatment in the elderly is especially challenging due to an increased prevalence of multi-morbidity along with changes in physiology, pharmacokinetics and pharmacodynamics [5,6]. Information or knowledge regarding medications is ever growing and needs to be continuously updated and implemented in clinical practice [7,8,9]. 

Clinical decision support systems (CDSSs) in the medication management process are being used to support decisions regarding medication, to facilitate evidence-based medicine, to reduce the incidence of DRPs and to improve health care quality and efficiency [7,10,11,12,13,14,15,16]. A CDSS can, for example, support physicians and pharmacists in detecting potential DRPs by linking patients´ current medications together with patient-specific factors. By using defined algorithms based on a knowledge database, analysis of these data will generate patient-specific alerts. Some medications are known to have an unfavourable risk–benefit balance for older patients and are often referred to as potentially inappropriate medications for the elderly [17]. CDSSs have been shown to be a useful tool to reduce potentially inappropriate medications in the elderly [17]. 

At community pharmacies, pharmacists are responsible for the safe dispensing of prescription drugs and for examining prescriptions before dispensing, and thus they play an important role in detecting prescription errors and preventing DRPs [18,19,20]. A pharmacy dispensing information system is a system used at pharmacies for processing and dispensing prescriptions, but systems differ in functionality and design. One way of supporting pharmacists in detecting potentially inappropriate prescriptions or avoiding dispensing errors are CDSSs that may be integrated in the pharmacy information system or provided in another manner [13,21,22,23,24]. 

At pharmacies in Sweden, a CDSS called Electronic Expert Support (EES) is available. EES analyses patients´ electronically stored prescriptions in the Swedish national prescription repository in order to detect potential DRPs such as drug–drug interactions, high doses, therapy duplications, and inappropriate drugs and doses for elderly patients or paediatric patients. 

EES is not available to prescribers in Swedish health care. Previous studies have described the potential DRPs detected by EES in patients with multi-dose drug dispensing and have shown that physicians regard the majority of alerts as clinically relevant [25,26]. Although EES has been available at pharmacies for many years, its use has been low and only studied in a few small studies where most of them are not published scientifically [27]. Behaviour change interventions and strategies for implementing clinical guidelines include, for example, education and the implementation of CDSSs, but the effects of these interventions vary [28,29]. The effects and the use of a CDSS depend on factors such as implementation, design, clinical relevance of alerts [9,30,31,32,33,34] and social factors [9,35]. Many alerts from CDSSs are being ignored, and the override rates range between 29% and 91% depending on the categories of alert [36,37,38,39,40]. 

A nationwide intervention initiated by the association of Swedish pharmacies was performed as a joint effort between all Swedish pharmacy chains in 2018 in order to increase the use of EES, focusing on elderly patients. The goal of the national intervention was to offer each patient aged 75 years or older, collecting prescription medication at a Swedish community pharmacy during one specific week, an EES analysis. The intervention included a web-based education module for pharmacists as well as information within each pharmacy company about the intervention week. It is not known if this kind of nationwide intervention involving several different pharmacy companies can have a significant effect on the use of IT applications in pharmacy practice. From previous research we know that it is important to pay attention to many different factors in relation to the use and implementation of health IT. Educating and giving instruction on how to use a system may not be enough if there are other issues that have to be improved or solved. A holistic sociotechnical approach is needed to better understand the use and implementation of health IT, involving both organizational, technical and user-specific aspects.

The aim of this research was to study the use of EES in connection with the national intervention in order to describe any effects of the intervention, to better understand how EES is used by pharmacists and to identify any barriers and facilitators for the use of EES by pharmacists as a part of their work to improve medication use for elderly patients. More specifically, we wanted to

- examine the impact of the national intervention on the weekly number of EES analyses;
- describe the proportion of patients aged 75 years or older getting an analysis with EES; the number of alerts for potential DRPs; and the proportion being resolved before, during, and after the intervention;
- describe what types of alerts for potential DRPs that were generated, which types were resolved, and what kinds of actions that were taken to resolve them during the week of the intervention; and
- describe pharmacists’ perceptions and experience with EES and the national intervention.

## 2. Materials and Methods 

The study had two parts and used a mixed methods approach, including both data on the use of EES before and after the national intervention and a questionnaire distributed to pharmacists at Swedish community pharmacies. A parallel design was used, where quantitative and qualitative data were collected concurrently and analysed separately. This approach, combining objective data on actual use in all of Sweden and subjective views from pharmacists using the system, provides insights and deeper understanding about both “how” and “why” [41].

### 2.1. Setting

In this nationwide study we included all pharmacies in Sweden, approximately 1500 pharmacies in total. To dispense a prescription, pharmacists in Sweden have to use a dispensing system in order to handle relevant information and tasks. When the study took place in 2018, Sweden had the following pharmacy chains; Apoteket AB, Apotek Hjärtat, Apoteksgruppen, Kronans Apotek, Lloyds Apotek and Apotea as well as some small independent pharmacies. All community pharmacies in Sweden are connected to the prescription repository, which includes 99% of the prescriptions being dispensed in Sweden (less than 1% being paper prescriptions or other prescriptions). 

#### 2.1.1. Electronic Expert Support System (EES)

EES is a government-owned CDSS that analyses patients´ electronically stored prescriptions in the Swedish national prescription repository. It was originally developed by Medco Health Solutions in the US and has been adapted to Swedish clinical practice [25]. EES is available to Swedish pharmacies and is developed and maintained by the Swedish eHealth Agency. EES has been available in pharmacies since 2010, and the level of use was initially low but has been increasing. EES has gone through extensive development since then and is continuously being updated. EES can detect potential DRPs, including drug–drug interactions (since 2016 based on Janusmed Interactions, previously known as Sfinx [42], the same interaction database as available in health care), therapy duplication (dispensing of two or more drugs within the same therapeutic category), high dose (a prescribed dose exceeding the maximum daily dose), drug–disease inferred (potential contraindications for a drug with an existing inferred disease), drug gender warning and potentially inappropriate drugs and doses for elderly patients or paediatric patients [25]. The alerts are visible to the pharmacists first when the pharmacists make an active choice to perform an EES analysis, which also requires patient consent the first time. In some pharmacy chains, EES is integrated into their dispensing system, and at some pharmacy chains the pharmacist accesses EES over the Internet using a separate web browser. Each time a pharmacist utilises EES (i.e., presses the button in the system), EES analyses the patient’s prescriptions and may generate a number of alerts. EES analyses current prescriptions in the prescription repository, as well as previous prescriptions for which it is likely that the patient still has medication left at home. When the pharmacist resolves a DRP, they can close the alert and document the reason so that it will not show again when the same medications are dispensed on another occasion.

#### 2.1.2. National Intervention

In connection with this study, a national intervention called “the focus week” was performed. The goal of the national intervention was to offer each patient aged 75 years or older collecting prescription medication at a Swedish community pharmacy during one week (week 15, 9 April to 15 April 2018) an analysis of the patient’s prescriptions in the prescription repository using EES in order to detect potential DRPs. During the intervention, the pharmacy chains in Sweden joined towards this goal, also setting the goal that all pharmacists at community pharmacies should complete the web-based education regarding the use of EES before the week of the intervention. The long-term aim for the intervention was to increase the knowledge and use of EES among community pharmacists in order to increase medication safety among the elderly. 

Before the week of the intervention, information was sent to the pharmacists by the respective pharmacy chain on several occasions informing them about the intervention and about the research being done in connection with the intervention. The first information was sent in January/February 2018. Information regarding the intervention and the research was also available to pharmacies and patients through other channels.

This nationwide intervention was the first of its kind (i.e., all chains joining in together) since the re-regulation of the Swedish pharmacy market. The association for pharmacies (Sveriges Apoteksförening) made the initiative for the intervention, and their board decided that all pharmacies in Sweden would participate. They were also the initiator of the research study, but were not involved in the research itself. 

### 2.2. Data and Statistics on The Use of EES

Data and statistics from the eHealth Agency included the number of EES analyses, the number of individuals having prescriptions dispensed, the number of EES alerts, the number of resolved alerts and the number of active pharmacies (Table 1). The data are automatically generated when EES is used, covering all pharmacies using EES in Sweden. Different data were available for different time periods and populations. Statistics on the use of EES for the total population were available per year from 2014 to 2018 and per week from 2017. Data specifically for individuals 75 years or older were only available for certain weeks. Week 15 of 2018 was the primary week for measurement, i.e., the week of the intervention. For comparison, we chose four other reference weeks during the year for which we collected data: one of the weeks was before the intervention (week 11 of 2018, which was as early as possible when data for patients 75 years or older were available) and three of the weeks were after the intervention (week 21 of 2018, week 36 of 2018, and week 11 of 2019). The reference weeks were chosen to be as representative as possible, i.e., no holidays or special events. Data on the number of pharmacies, types of alerts for potential DRPs that were generated, which alerts were resolved and what kinds of actions were taken to resolve them were also provided for the week of the intervention. All data were extracted and handled in an aggregated form, and no data pertaining to individuals were used by the researchers.

### 2.3. Questionnaire among Pharmacists

The web-based questionnaire comprised 20 questions divided onto six pages and was developed by the researchers for the study. The questions included multiple-choice questions, statements where the respondents gave their degree of agreement with a statement according to a six-point Likert scale, and open-ended questions that could be answered in free text (Table 2). The face validity of the questionnaire was evaluated by experts in the reference group for this study. The questionnaire was then tested among 13 individuals, most of whom were pharmacists, to see if the questions were relevant and easy to answer from their perspective. The questionnaire was then slightly adjusted before the large-scale survey.

The questionnaire was sent to the work-related email addresses of pharmacists randomly selected from all pharmacies in Sweden. All pharmacy chains in Sweden were included in the study, and the lists of email addresses were provided by the respective pharmacy chains. A random selection using the RAND function in Excel was made by the researcher (TH) based on the proportion of the pharmacy market each pharmacy chain had in January 2018. For most of the pharmacies, including the largest ones, the random selection was made from a list of all pharmacists employed by the pharmacies. However, exceptions had to be made for some of the pharmacies. For one pharmacy chain, the random selection had to be made from a list of the pharmacists in charge of quality management. For the small independent pharmacies and one chain the questionnaire was instead sent to a shared email address for each of the individual pharmacies where it could be answered by one of their pharmacists. Along with the questionnaire, the pharmacist received information about the research, about the questionnaire and how the data would be used and handled. By answering the questionnaire, the pharmacists agreed to be included in the study. 

The questionnaire was distributed to pharmacists the week after the intervention. The questionnaire was sent to 1500 pharmacists at Swedish pharmacies, and the responses were collected from 17 April 2018 to 27 May 2018. From a total population of pharmacists of approximately 5000 individuals, it was calculated that at least 357 answers to the questionnaire were needed in order for our sample to represent the views of pharmacists in Sweden in general with a margin of error of 5% and a confidence level of 95%. The questionnaire was originally sent to 1000 pharmacists, but after two weeks with one reminder the number of responses was lower than expected (n = 254), thus another 500 pharmacists were randomly selected following the same procedure as the first 1000 and were sent the questionnaire on 3 May 2018. After that, two reminders were sent out to all pharmacists included in the study, thus the first 1000 received a total of three reminders and the 500 included later received two reminders. 

### 2.4. Data Analysis

Data was analysed using Excel, SPSS and STATA. Analysis of the effects of the intervention on the number of EES analyses was performed as an interrupted time series analysis. In the first step, a two-sample t-test with unequal variances was used to present before and after intervention comparisons. In the next step, a single interrupted time series analysis (ITSA) was conducted to evaluate the intervention effect. This regression approach included three covariates. The first covariate (t) represented the pre-intervention slope and reflected the trend before the intervention. The second covariate (x) represented the change in the level at the intervention point, i.e., the level change between the time points immediately before and after the intervention. The third covariate (xt) represented the change in the slope from pre-intervention to post-intervention [43]. In addition to these three covariates, a post-intervention slope was calculated. The number of EES analyses each week for the total population was used as the outcome variable in the regression model. In total, the data included 115 time points (weeks), and the intervention was conducted at week 67. Problems with autocorrelations (i.e., that a measure at some time point is correlated with its past values) were identified by the Breusch–Godfrey (x2(1) = 56.7, p < 0.001) [44]. To handle this problem, OLS regression with Newey–West standard errors were used. The number of lags was based on four goodness-of-fit indices that all suggested three lags: Akaike Information Criterion (AIC), Bayesian Information Criterion (BIC), Final Prediction Error (FPE) and Hannan–Quinn Information Criterion (HQIC). The interrupted time-series analysis was performed in Stata 16.0, including the command ITSA [45].

The data from the questionnaire were treated as ordinal data and analysed with descriptive statistics in SPSS. The responses to the questionnaire in free text were analysed and categorised using manifest content analysis methods [46]. 

### 2.5. Ethics Statement

The study was conducted in accordance with the Declaration of Helsinki, and the protocol was assessed by the Ethical Advisory Board in South East Sweden. We received an advisory ethical assessment from the Ethical Advisory Board in South East Sweden (Date 19 February 2018, Project identification code EPK 469–2018, *Advisory Opinion on the project “Pharmacists’ use of Electronic Expert Support (EES) to improve drug use for the elderly”*). Based on the nature of the data being handled and collected the Ethical Advisory Board did not see any ethical issues or obstacles with the present study. The Ethical Advisory Board did not regard the research to fall within the Swedish Ethical Review Act (SFS 2003:460); and accordingly, an additional ethical approval from the regional ethics committee was not necessary. The national intervention at pharmacies was a part of the pharmacies’ improvement work and was not a part of the research. The researchers did not initiate or control the intervention, and only measured any effects of it. The data from the Swedish eHealth Agency were delivered to the researchers in aggregated form with no data on the individual level. The questionnaire for the pharmacists only asked about their professional role and was conducted and analysed in a de-identified form so that it was not possible to link individual pharmacist’s identities to their responses.

## 3. Results

**Figure 1:**
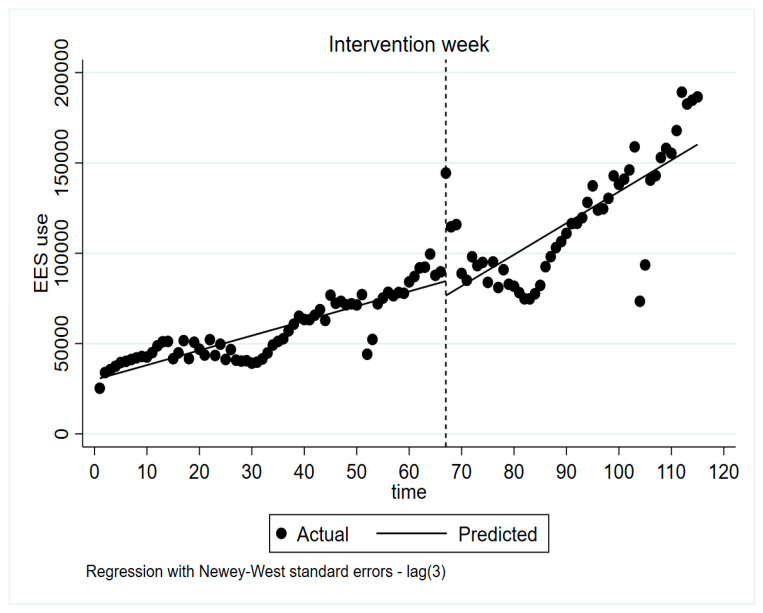
Number of Electronic Expert Support (EES) analyses per week between week 1, 2017 and week 11, 2019. Interrupted time series regression of the use of EES (number of EES analyses) for the total population, comparing the trend before and after the intervention. The time series starts week 1, 2017 (time = 0), and ends week 11, 2019 (time = 115). The intervention occurred during week 15 in April 2018 (time = 67), indicated as a dotted line. The four reference weeks used in this study was week 11 of 2018 (time = 63), week 21 of 2018 (time = 73), week 36 of 2018 (time = 88), and week 11 of 2019 (time = 115). Summer holidays in Sweden occurred during June to August (primarily during time = 24–33 and time = 76–85), and Christmas holidays occurred in the end of December (primarily during time = 51–53 and time = 102–105) Regression with Newey–West standard errors. Number of observations = 115. Maximum lag = 3. Performed with the ITSA script for STATA.

The use of EES, i.e., the number of EES analyses, has increased since it was implemented. In 2014, the number of analyses was 125,637 compared with 5,190,519 in 2018. The number of EES analyses per week for the total population was 25,278 in week 1 of 2017 and 186,557 analyses in week 11 of 2019, which represents an approximately sevenfold increase (Figure 1). The number of EES analyses in the week of the intervention for the total population was 144,423.

### 3.1. Effects of The National Intervention on The Use of EES 

The number of EES analyses per week was significantly higher after the intervention (mean = 117,860, number of observations = 48) compared with before the intervention (mean = 58,568, number of observations = 67, p = 0.000) (Figure 1). Before the intervention, the increase in the number of EES analyses every week (the pre-intervention slope) was on average 816.0 analyses (95% CI 652.5–979.5). After the intervention, there was an average increase in EES analyses (the post-intervention slope) of 1742.8 (95% CI 884.2–2601.5) per week. Thus, the increase per week was significantly higher after the intervention compared with before (Coef. 926.8, 95% CI 38.4–1815.1, p = 0.041) as seen by the change in slope (Figure 1). The time series plot indicates that the level of use decreased markedly during holidays both before and after the intervention, especially during summer and Christmas. 

### 3.2. EES Analyses and The Numbers of Alerts Generated and Resolved before and after The Intervention

For patients aged 75 years or older, the number of EES analyses the week of the intervention was 55,180, which represents 21% of those having prescriptions dispensed that week (Table 3). Alerts that were closed in the system represented almost 7% of the alerts. Both the numbers and proportions of EES analyses as well as closed alerts varied during the five weeks of measurements. 

The most common type of alert for a potential DRP among patients aged 75 years or older during the week of the intervention was drug–drug interactions (69% of all alerts) (Table 4). Different categories of alerts were resolved to different extents. More than 80% of the alerts being closed were due to dialogue with patient for verification of the treatment (Table 5).

### 3.3. Pharmacists’ Perceptions of EES and The Intervention

A total of 457 pharmacists working in Swedish pharmacies answered the questionnaire (response rate 30%, 457/1500). For background information of the respondents, see Table 6. Almost all (97.6%) of the pharmacists said that they had some form of education related to EES (Table 7), where the most common answer was a web-based course distributed by the Swedish eHealth Agency. The majority (58.9%) of the respondents said they had used EES daily during the past year (Table 7). Among the respondents, 99% (n = 450) knew about the national intervention during week 15 of 2018. Two-thirds of the respondents (n = 303) answered that their work had been affected during the intervention, 70% (n = 320) reported that they had used EES more actively overall, 74% reported that they used EES more than usual for pharmacy customers aged 75 years or older and 11% reported that they used it less than usual for pharmacy customers younger than 75 years. 

**Figure 2:**
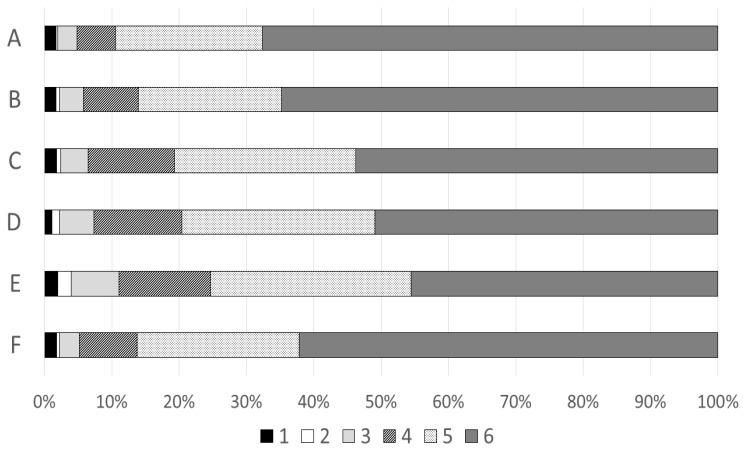
Perceptions regarding the use of EES among pharmacists on a six-point Likert scale (1 = do not agree at all, 6 = totally agree) (n = 457). The statements were as follows. (A) “EES provides support in improving the use of medications for patients aged 75 years or older.” (B) “EES provides support in improving the use of medication for patients younger than 75.” (C) “EES alerts are relevant to me when meeting the patients 75 or older.” (D) “The information presented in the alert description text in EES provides a good support for me in dialogue with patients aged 75 or older.” (E) “The information presented in the message text in EES provides a good support for me in dialogue with doctors for patients aged 75 or older.” (F) “I develop my pharmaceutical skills by using EES for patients 75 or older.” Missing answers ranged from 3 to 13 for the different statements and are not shown in the figure.

The proportion of pharmacists who agreed with the statements (answering with 4, 5 or 6 on the six-point Likert scale) ranged from 88% to 95% (Figure 2). The majority answered with 6 (totally agree) for five of the six statements. When the pharmacists were asked which EES alert category gave them the best support, the most common answer was drug-drug interactions (92%) (Table 7). The most common reason (73%) for not using EES to analyse prescriptions for patients 75 years or older was that it was difficult when medications were collected by a relative or caregiver (Table 7). Other reasons described in free text responses included, among others, lack of time at the pharmacy, the pharmacist forgetting, the patient not wanting it and difficulties related to communication. When asked what they would need to use EES more often, the most common answers were more time with the patient (71%), more knowledge among patients about EES (32%) and more experience (31%) (Table 7). The most common issue described in free text was that the pharmacists wished that they did not need consent to perform the EES analysis. Other reasons described in free text included examples of improvement or development of EES and more education for pharmacists. 

Pharmacists’ free text answers about their perceived need for improvement or additional functionality in EES revealed several categories/themes among the answers. Many pharmacists wanted to be able to add medication for analysis such as over-the-counter (OTC) medication. Many also wanted more alerts and new functionality to support them or allow them to access more information. Pharmacists also described a need for improvements of the system, and recurring answers were regarding the design/user interface, reducing the number of duplicate alerts, improving existing alerts, making it easier to close an alert, improving the documentation in the system, to have more education for pharmacists, and to remove the request for consent.

When the pharmacists were asked if they had ever handled or taken actions to prevent a potential problem according to an EES alert without closing the alert, many answered “yes, often” (38%) or “yes, sometimes” (47%) (Table 7). Among reasons for not closing an alert (among multiple choice alternatives), the most common answer was lack of time and that they wanted other pharmacists to see the alert. Free text answers about reasons for not closing an alert included a perceived unfinished task/matter, taking too much time, technical issues when trying to close an alert, that they forgot and uncertainty about the action or other reasons.

The pharmacists used many other decision support and information sources, including knowledge and support available online or in print. What pharmacists described in free text about their need in order to improve the use of medications included, among other things, more time for patients at the pharmacy, improved communication and connection with health care, improvements and development of the pharmacy information system or EES, more education for pharmacists, removing the need for consent from patients to perform an EES analysis, a different focus for the work at pharmacies and improvements related to generic substitutions. Other comments from the pharmacists were that they appreciated the intervention (the focus week) and wanted more of similar activities.

## 4. Discussion

This study shows that during the week of the intervention the number of EES analyses increased; approximately 21% of patients aged 75 years or older, and 15% of patients all ages, collecting prescription medications during the week of the intervention had their prescriptions analysed with EES. Furthermore, the results show that the mean number of analyses per week as well as the increase in number of analyses per week was significantly higher after the intervention compared with before. The questionnaire among pharmacists also showed that pharmacists appreciate EES and feel that it supports them in their work. Pharmacists described usability issues with the system, barriers for using EES, and had some suggestions for improvements. 

### 4.1. Effects of The National Intervention

This study shows that it seems possible to perform this kind of nationwide intervention involving all pharmacy companies, with a clear effect on pharmacists’ use of EES both short term and long term. Most pharmacists reported that they knew about the intervention and that their work was affected by it. Almost 90% of pharmacies in Sweden actively used EES during the week of the intervention. Moreover, the statistics on the number of EES analyses showed a marked increase during that specific week, and the results also indicated that the intervention contributed to a more rapid increase in the use of EES during that year after the intervention compared with before. The interrupted time series regression did not show a level increase, only a slope increase. The reason for this is not clear but it is probably partly due to the summer holidays starting soon after the intervention. Data on the use of EES from previous year’s show that the use of EES always decreases markedly during June to August. Moreover, for a sustainable increase a more long-term change in working procedures may be necessary which perhaps took some time. The pharmacies’ goal with the intervention was to provide all patients aged 75 years or older with an EES analysis. A potential negative effect of this kind of focused intervention could have been that other groups got less attention, but our results show the opposite. 

As far as we can find, there are no similar studies on the effects of interventions at pharmacies to increase the use of CDSSs. Behaviour change interventions including, for example, education, training or implementation of CDSSs have been shown to effectively modify healthcare professionals’ practice and patients’ outcomes; however, the effects of such interventions vary [29]. A systematic review of intervention research to enhance community pharmacists’ cognitive services showed that planned interventions have the potential to improve and expand pharmacists’ cognitive service delivery, but that there is a need for well-designed intervention research that can evaluate the impact of such interventions [47]. A systematic review of implementation strategies for clinical guidelines to community pharmacies showed that the most commonly used strategy was education, but the greatest effect on outcome was demonstrated using CDSSs [28]. In the present study, the CDSS was already in place and the intervention was instead directed at increasing the use.

From previous research we know that organisational aspects are very important for successful implementation of health information technology [27,48,49]. This intervention involved pharmacies at the management level, which can have long-term effects on leadership and other organisational aspects. All these aspects might have contributed to the effect of the intervention. Moreover, because preparation for the intervention started already during the fall/winter 2017, this could have contributed to an increase in the use of EES before the intervention as well. 

This study shows that the use of EES varied over time and that the use decreased markedly during summer and Christmas. The system is continuously being updated and developed, and the alert algorithms are adjusted such that new alerts might be added or removed, which might affect the number of alerts being generated and in turn might affect the proportion of alerts being resolved. For example, although the absolute number of alerts being closed was higher during week 11 of 2019 compared with week 11 of 2018, the proportion of alerts being closed was lower. In 2019 each EES analyses generated a higher number of alerts than before. Our results indicate that perhaps pharmacists do not have enough time to handle all the new alerts, or more of the alerts were not relevant. Other types of designs or integration into dispensing systems can also affect how and when EES is used. This finding highlights the importance of measurements over time when studying the use of a CDSS. 

### 4.2. Alerts Being Resolved

Only a small proportion of alerts were being closed. However, pharmacists’ responses to the questionnaire showed that it was common that they resolved issues related to an alert without closing the alert. The most common reasons for this were a lack of time and that they wanted other pharmacists to see the alert. Our results indicate the need for improvement of the system so that it is easier to close an alert and perhaps finding other ways of documenting or communicating between pharmacists related to an alert.

The proportion of alerts being closed differed between alert categories indicating that pharmacists acted more often on potentially more serious alerts. One explanation can be that pharmacists can filter for alerts in their system, for example choosing not to see A- and B-interactions (less serious). Not closing alerts for A-interactions also indicate that a lack of time is a major obstacle or that is unclear when to close the alert. Tracking the closing of non-relevant alerts could be used to further improve the system and reducing the risk of alert fatigue. 

The way EES is currently implemented and regulated, the pharmacists have to make an active choice to perform an EES analysis, which is different from many other CDSSs that are integrated and automatically provide alerts or indicate that there might be a potential DRP. This makes it difficult to compare our results with the numbers of alert overrides from other studies. 

### 4.3. Pharmacists’ Perceptions and Experiences with EES 

Previously reported barriers to implementing permanent therapy changes based on CDSSs include lack of time, low alert specificity, and poor agreement and communication between health care services and pharmacies [17]. All these aspects were also mentioned in free text by pharmacists in different parts of the questionnaire, and many stated that they wanted to reduce the number of duplicate alerts or improve existing alerts. Other more specific issues or barriers found in this study include not having enough experience and difficulties when the patient does not come to the pharmacy themselves, but rather a relative or caregiver is picking up the medication. Pharmacists also wanted to be able to add non-prescription medications to the analysis, something that is not possible today. At the time of the study, patient consent was needed to perform an EES analysis, even though the pharmacists automatically view all of a patient’s prescriptions when a patient wants to collect prescription medication at a pharmacy. The need for this extra patient consent to perform an EES analysis was repeatedly mentioned as problematic in this study, as well as in a previous study [27]. Moreover, the second most common suggestion for improvement was that patients should have more knowledge about EES. Having to explain the system to obtain consent was perceived as time consuming and confusing for patients. A study among pharmacy customers in 2018 indicate that their knowledge and awareness of pharmacists using EES is low, but that they seem to be generally positive towards this kind of review of prescriptions (unpublished data under review, Hammar et al.). The requirement for consent was reassessed by the Swedish eHealth Agency from a legal point of view after this study was conducted, and the need for the consent in order to perform an EES analysis was removed in June 2020.

### 4.4. Using CDSSs to Improve Medication Safety

From this study we cannot draw any conclusion on clinical effects from the use of EES, neither on clinical relevance of the alerts. Theoretically, increased use of a CDSS would indicate that pharmacists are working with decreasing the risk of potential DRPs. However, we cannot know what actions would have been taken regardless of the availability of the CDSS. Neither do we know if it would be reasonable to perform EES analysis for all patients or if it would be reasonable to take actions and resolve all of the alerts generated by EES. Some of the alerts may already have been resolved or evaluated by the healthcare services without the pharmacists knowing about this, and there is a risk that patients receive contradicting information from the pharmacist. A previous study among physicians showed that they regarded 68% of EES alerts to be clinically relevant, and 11% of all alerts were followed by a change in drug treatment [26]. In our study, contact with the prescriber and change of prescription was documented for only 0.2% of all closed alerts. Even if the true rate might be higher (but alerts not closed), we cannot say if there were alerts overridden by the pharmacist that would have been regarded as clinically relevant by the prescriber. Pharmacists do not have access to patient data from electronic health records (EHR) such as medication history, diagnoses and lab values. This contributes to some difficulties in deciding if the alert is clinically relevant. Although pharmacies and health care cannot use the same decision support, it would be good if the knowledge database used in their respective CDSS were the same so that the alerts they receive correspond with each other, like with the interaction database used today on both health care CDSS and EES. In Sweden, prescribers do not have access to the Swedish national prescription repository and pharmacists at community pharmacies do not have access to the medication list in the EHR. Thus, having access to different lists of a patient’s current medications can also lead to pharmacists and physicians receiving alerts that are not the same. A new nationally shared medication list is being implemented based on a new law put into force in 2021 with the goal that all of the involved actors will have access to the same medication list [50]. Hopefully, this can contribute to improved accuracy of the medication lists, improved relevancy of CDSS alerts, as well as facilitating collaboration between health care and pharmacies [51,52].

Some unintended consequences of the implementation of CDSSs have been described, for example, increased pharmacist–physician communication load [53]. Research has also shown the need for improvement of many aspects of CDSSs such as usability [40].

It is important for quality work at pharmacies to get knowledge about the detection and handling of potential DRPs. Another study described the development and evaluation of a coding to systematically collect and assess information on pharmacists’ interventions [54], and in that study the documentation was manual. 

Increasing the use of a CDSS and preventing DRPs likely requires organisational interventions, such as solving issues with pharmacists not having enough time to deal with the alerts, as well as optimising the design of the system and the clinical relevance of the alerts. Other changes that might be good to consider include other means of documenting or communicating in relation to the alerts.

### 4.5. Method Discussion (Strengths and Weaknesses)

The strengths of the study were the national perspective, covering all pharmacies in Sweden, and the combination of methods, with objective and automatic measurements of CDSS use at different points in time together with questionnaire data. The data from the Swedish eHealth Agency regarding dispensed prescriptions and the use of EES are automatically generated for statistical purposes and are of high quality. However, the study was not designed to identify effects on any clinical or patient outcomes.

The questionnaire had a response rate of 30%, which is in line with other similar studies. The pharmacists answering the questionnaire may have been more positive towards EES and been using it to a higher degree than pharmacists in general. This is indicated by data from the eHealth Agency showing a lower use than indicated by the pharmacists’ responses on the questionnaire regarding how often they used EES. The response rate and the use of EES also varied between different pharmacy chains. The selection of the pharmacists could not be made randomly at some of the smaller pharmacy chains, thus the results from the questionnaire may not be possible to generalise to all pharmacists in Sweden. 

Because the use of EES has been continuously increasing, it is difficult to separate the effect of the intervention from the increase that would have occurred anyway. The interrupted time series regression comparing the slope before the intervention to that after the intervention shows a faster increase; however, we cannot be sure that this long-term increase would not have occurred independent of the intervention.

### 4.6. Future Research

Community pharmacy practice as well as the pharmacist’s role is changing due to several factors, including digitalisation, new pharmacy services and increased knowledge. It is important therefore to perform research in community pharmacies as well as to develop strategies for how to implement new knowledge in practice [55]. In Sweden, pharmacy research decreased after the re-regulation of the pharmacy market. The current study described an intervention as a joint effort between all pharmacy chains in Sweden, which is a positive development. Future studies should investigate the clinical effects of the use of CDSSs and how the use of CDSSs is affected if the requirement for consent is removed or if EES is integrated in other ways in the dispensing systems.

## 5. Conclusions

This study shows that pharmacists use and appreciate EES as a decision support at pharmacies and that the national intervention had a clear effect across different pharmacy chains during the week of the intervention and seems to have contributed directly or indirectly to a faster increase in pharmacists’ use of EES during the year to follow. However, the study also identified several barriers or issues that need to be addressed to facilitate an increase in the use of the system, such as the usability of the system, specificity of the alerts, clarification about when to close an alert, the requirement for patient consent and the lack of time. The effects seen by the intervention are likely multifactorial, through the education and information directly to pharmacists, but probably also on an organisational level because the pharmacy chains were involved in the intervention on a higher management level, which might have affected leadership, routines and other organisational factors. More research is needed on the actual clinical effects of using EES and strategies for increasing the use of CDSSs at pharmacies.

## Figures and Tables

**Table 1:** Description of data and statistics, time period and population. If not stated otherwise, data are for both the total population and specifically for individuals aged 75 years or older at the time.

Data	Description	Time Period
Number of EES analyses	*Statistics from eHealth Agency* Number of times EES is used, active choice to press the EES button. (only calculated once/unique individual/pharmacy/day).	*Total population* Per year 2014–2017 Per week from week 1 of 2017 to week 11 of year 2019.
Number of EES analyses	*Statistics from eHealth Agency* Number of times EES is used, active choice to press the EES button. (only calculated once/unique individual/pharmacy/day).	*75 years or older* Per week (week 11,15, 21, 36 of 2018 and week 11 of 2019)
Individuals having prescriptions dispensed	*Statistics from eHealth Agency* Number of individuals having prescriptions dispensed (only calculated once /unique individual/pharmacy/day).	Per week (week 11, 15, 21, 36 of 2018 and week 11 of 2019)
Proportion of individuals getting an EES analysis	*Calculation* Number of EES analyses/Individuals having prescriptions dispensed (%).	Per week (week 11, 15, 21, 36 of 2018 and week 11 of 2019)
Number of EES alerts	*Statistics from eHealth Agency* Total number of alerts from EES. Each time a pharmacist utilises EES, EES analyses the patient’s prescriptions in the prescription repository and may generate a number of alerts.	Per week (week 11, 15, 21, 36 of 2018 and week 11 of 2019)
Average number of alerts per EES analysis	*Calculation* Number of EES alerts/number of EES analysis. How many alerts are generated on average each time EES is used.	Per week (week 11, 15, 21, 36 of 2018 and week 11 of 2019)
Closed (resolved) EES alerts	*Statistics from eHealth Agency* In EES the pharmacist can close an alert after it has been resolved. When an alert is closed the pharmacist can provide the reason for closing the alert from a number of alternatives in the system.	Per week (week 11, 15, 21, 36 of 2018 and week 11 of 2019)
Proportion of alerts being closed	*Calculation* Closed alerts/number of alerts (%).	Per week (week 11, 15, 21, 36 of 2018 and week 11 of 2019)
Type of alert generated, resolved, and documented action	*Statistics from eHealth Agency* According to eHealth Agencies alert categories and reasons for closing alert available in the system.	Week 15 of 2018
Active pharmacies	*Statistics from eHealth Agency* Number of pharmacies actively using EES, e.g., at least one EES analysis during the week.	Per week (week 11, 15, 21, 36 of 2018 and week 11 of 2019)

**Table 2:** Description of the questionnaire developed for the study. For multiple-choice questions, the alternatives can be seen from Figure 2 and Tables 6 and 7.

	Question	Description
1	Year of birth	YYYY
2	Gender	Multiple choice:
3	Education	Multiple choice
4	Years as a pharmacist at a community pharmacy	Multiple choice
5	What pharmacy chain do you work at?	Multiple choice
6	How do you characterise the vicinity of the pharmacy where you most often work?	Multiple choice
7	How do you characterise the city or region where the pharmacy where you most often work is located?	Multiple choice
8	How often have you on average used EES during the past year?	Multiple choice
9	Five questions about the intervention (here called focus week) *Have you received information about the focus week (before this questionnaire)?* *Has your work been affected by the focus week?* *Have you used EES more actively in general during the focus week?* *Have you used EES more actively for patients aged 75 years or older during the focus week?* *Have you used EES less than usual for patients younger than 75 years during the focus week?*	Multiple choice: yes/no/do not know *Before the questions, the questionnaire had one sentence about the focus week for clarification*
10	Have you gone through any education regarding EES? *(Web-based education from eHealth Agency/Education with a supervisor from eHealth Agency/Meetings or days of education at your pharmacy chain/ Reading material on your own/Other education/ No education/ Do not know)*	Multiple choice Several answers possible. Possible to answer in free text
11	Six statements about the pharmacist’s perceptions about using EES	Degree of agreement on a six-point Likert scale (1 = do not agree at all, 6 = totally agree)
12	Which EES alert category do you perceive provides you with the best support in pharmacological control (for pharmacy customers all ages)	Multiple choice. Several answers possible.
13	Have you ever handled or taken actions related to an EES alert, for example, having a dialogue with the customer or contacting the prescriber, without closing the alert?	Multiple choice
14	If you answered yes to 13, what was the reason?	Multiple choice. Several answers possible. Possible to answer in free text.
15	For the times you do not use EES for pharmacy customers aged 75 years or older, what is the reason?	Multiple choice. Several answers possible. Possible to answer in free text.
16	What would you need to use EES more often?	Multiple choice. Several answers possible. Possible to answer in free text.
17	Do you perceive any needs for improvement or development in EES? Describe these.	Free text
18	What other decision support or sources of information do you use regularly (at least once a month) in your work to improve use of medication?	Multiple choice. Several answers possible. Possible to answer in free text.
19	What would facilitate or make it easier for you in your work with improving the use of medications for pharmacy customers aged 75 years or older?	Free text
20	Do you have any other comments about EES, the focus week, or the questionnaire?	Free text

**Table 3:** Number and proportion of EES analyses and alerts being resolved during the week of the intervention (week 15) as well as the reference weeks before and after. Patients aged 75 years or older and patients of all ages (total population). For details on data and calculations, see Table 1.

		Week 11 2018	Week 15 2018	Week 21 2018	Week 36 2018	Week 11 2019
**75 years or older**	Number of EES analyses	27,829	55,180	28,348	31,635	51,953
	Individuals having prescriptions dispensed	253,938	257,783	258,449	253,678	261,990
	Proportion of individuals getting an EES analysis	11%	21%	11%	12%	20%
	Number of EES alerts	139,648	329,138	137,708	207,583	339,289
	Average number of alerts per EES analysis	5.0	6.0	4.9	6.6	6.5
	Number of closed EES alerts	4601	21,952	3579	3662	6327
	Proportion of alerts being closed (%)	3.3%	6.7%	2.6%	1.8%	1.9%
**Total population**	Number of EES analyses	92,312	144,423	93,175	103,153	186,557
	Proportion of individuals getting an EES analysis	10%	15%	10%	11%	19%
	Number of EES alerts	587,323	837,572	585,236	630,909	1,104,063
	Average number of alerts per EES analysis	6.7	5.8	6.3	6.1	5.9
	Number of closed EES alerts	14,906	46,634	12,606	11,715	23,620
	Proportion of alerts being closed (%)	2.5%	5.6%	2.2%	1.9%	2.1%
	Number of active pharmacies	1245	1358	1301	1342	1390

**Table 4:** Number of alerts per category, number of closed alerts, and proportion of alerts being closed within each category (week 15, patients aged 75 years or older). Explanation of alert categories: A interaction (Minor interaction of no clinical relevance), B interaction (Clinical outcome of the interaction is uncertain and/or may vary), C interaction (Clinically relevant interaction that can be handled, e.g., by dose adjustments.), D interactions (Clinically relevant interaction. The combination is best avoided.), therapy duplication (dispensing of two or more drugs within the same therapeutic category), high dose (a prescribed dose exceeding the maximum daily dose), high dose for elderly (a prescribed dose exceeding the maximum daily dose for elderly) drug–disease inferred (potential contraindications for a drug with an existing inferred disease), drug gender warning, and supplementary rules (alerts outside the other categories such as potentially inappropriate combination of drugs for elderly patients).

Alert Categories	Number of Alerts	Proportion of All Alerts (%)	Number of Closed Alerts	Proportion being Closed (%)
B interaction	103,722	31.5	1828	1.8
C interaction	97,710	29.7	9040	9.3
Therapy duplication	54,512	16.5	5843	10.7
High dose for elderly	19,716	6.0	1948	9.9
A interaction	17,231	5.2	37	0.21
Supplementary rules	8226	2.5	428	5.2
D interaction	7140	2.2	1107	15.5
Drug-disease inferred	6714	2.0	464	7.0
High dose	2680	0.8	187	7.0
Drug gender warning	74	0.0	6	8.1
Total	329,138		21,952	6.7

**Table 5:** Action to resolve an alert (i.e., reasons for closing an alert documented in the system, number for each category (week 15, patients aged 75 years or older).

Reason for Closing Alert	n	%
Dialogue with patient—verification of treatment	18,055	82.2
Only pharmaceutical assessment	1989	9.1
Dialogue with patient—referring to prescriber	869	4.0
Dialogue with patient—maculation of prescription	255	1.2
Contact with prescriber—without change of prescription	143	0.7
Contact with prescriber—change of prescription	44	0.2
Other action/measure	597	2.7
Total	21,952	100

**Table 6:** Background information of respondents (n = 457).

Background Characteristics		n	%
Gender	Female	398	87.1
	Male	55	12.0
	Other	4	0.9
Year of birth	1959 or before	66	14.4
	1960–1969	100	21.9
	1970–1979	125	27.4
	1980–1989	125	27.4
	1990 or later	41	9.0
Education *	Pharmacist (5–year education)	157	34.4
	Pharmacist (or prescriptionist, 3–year education)	297	65.0
	Other	3	0.7
Years as pharmacist at a community pharmacy	< 5 years	99	21.7
	5–10 years	123	26.9
	11–20 years	127	27.8
	> 20 years	108	23.6
Pharmacy chain	Apotea	10	2.2
	Apotek Hjärtat	178	38.9
	Apoteket AB	124	27.1
	Apoteksgruppen	61	13.3
	Kronans Apotek	68	14.9
	Lloyds Apotek	11	2.4
	Other pharmacy company	5	1.1
Pharmacy area/vicinity	Pharmacy in connection with hospital	39	8.5
	Pharmacy in connection with primary healthcare centre	107	23.4
	Pharmacy in connection with commercial streets/major shopping mall/supermarket	230	50.3
	Pharmacy in connection with junction/public transport	36	7.9
	None of the options above	45	9.8
City or region where the pharmacy is located	Larger city (more than 100,000 inhabitants)	141	30.9
	Small town (10,000–100,000 inhabitants)	229	50.1
	Village/sparsely populated region (< 10,000 inhabitants)	80	17.5
	None of the options above	7	1.5

* In this study, we use the term pharmacist when referring to the two categories of licensed pharmacists in Sweden: the pharmacists with 5 years of university education and prescriptionists with 3 years of university education. Both are licensed pharmacy practitioners with similar legal rights and obligations.

**Table 7:** Pharmacists’ answers to the questionnaire (n = 457).

Question	Answer (Multiple Choice)	n	%
How often have you used EES during the past year?	Daily	269	58.9
	Several times a week	132	28.9
	Several times a month	32	7.0
	A few times	20	4.4
	Never	2	0.4
	Do not know	2	0.4
Have you had any education regarding EES?	Yes	446	97.6
	No	11	2.4
Which alert category gives you the best support in the pharmaceutical control? **	Drug—drug interactions	421	92.1
	Elderly warnings	258	56.5
	Paediatric warnings	253	55.4
	High dose	237	51.9
	Drug—disease inferred	213	46.6
	Therapy duplication	197	43.1
	Drug gender warning	45	9.8
	Do not know	5	1.1
What are the reasons for not using EES to analyse prescriptions for patients 75 years or older? **	Difficult with relatives or caregivers collecting the medication	335	73.3
	The requirement for consent is an obstacle	209	45.7
	I feel that it takes too much time	144	31.5
	I see no need	133	29.1
	I do not have enough experience with the system	19	4.2
	I lack sufficient knowledge	10	2.2
	I think EES is difficult to use	6	1.3
	Other reason *	72	15.8
	Do not know	9	2.0
What do you need to use EES more? **	More time for the patient	326	71.3
	More knowledge among patients about EES	146	31.9
	More experience	141	30.9
	More user-friendly system	74	16.2
	Changed working procedures at the pharmacy	61	13.3
	Other functionality in EES	43	9.4
	More education	43	9.4
	Different leadership/management at the pharmacy	16	3.5
	Nothing, do not want to use	2	0.4
	Other *	37	8.1
	Do not know	19	4.2
Have you ever resolved a potential problem from an EES alert without closing it?	Yes, many times	172	37.9
	Yes, a few times	215	47.4
	No, never	48	10.6
	Do not know	19	4,2
If you have (*resolved without closing*), what is the reason for not closing the alert?	Lack of time	270	59.1
	Want other pharmacists to see the alert	187	40.9
	Not sure I selected/chose the right action	70	15.3
	Unclear when to close alerts	54	11.8
	Have not seen the meaning	16	3.5
	Other reason *	51	11.2
	Do not know	21	4.6

* Those who answered “other” could comment in free text; ** Several answers were possible.
